# Assessing thermo-physiological effects of different tree species within the inner regions of the urban canyon; confronting in-situ extreme heat stress in Istanbul during the El-Niño summer of 2023

**DOI:** 10.1007/s00484-025-02922-7

**Published:** 2025-04-23

**Authors:** Elif Nur Sarı, Andre Santos Nouri, Mert Ekşi, Andreas Matzarakis

**Affiliations:** 1 Department of Landscape Architecture, Faculty of Forestry, Istanbul University-Cerrahpaşa, Istanbul, Türkiye; 2 Chair of Environmental Meteorology, Faculty of Environment and Natural Resources, Albert-Ludwigs-University, D-79085 Freiburg im Breisgau, Germany; 3 Marine and Environmental Sciences Centre—MARE/Associate Laboratory ARNET—Aquatic Research Network, Department of Environmental Sciences and Engineering, NOVA School of Science and Technology—NOVA FCT, NOVA University Lisbon—UNL, Campus de Caparica - 2829–516, Caparica, Portugal; 4 Democritus University of Thrace, Komotini, Greece

**Keywords:** Urban typo-morphology, Istanbul, Heat Stress, Tree species, El Niño

## Abstract

This study investigates the impact of street morphology and tree species on thermal comfort in Istanbul during the July 2023 El Niño event, focusing on worst-case scenarios. Field measurements were conducted in the most common street morphologies and compared with data obtained from meteorological stations (MS). Subsequently, the influence of tree presence were evaluated for the measured streets, and PET assessments were conducted by incorporating fisheye photographs of the most common tree species in the region into the SVF calculations. The results indicate that EW-oriented streets, particularly the Left Lateral, experience beyond extreme heat stress due to extended sun exposure. PET results from MS were inconsistent with local conditions. The analysis of *Platanus orientalis*, *Populus canadensis*, and *Robinia pseudoacacia* on PET in different street orientations showed reductions of 5–6 °C, particularly for the first two species during morning and midday. This equates to a PET reduction from Beyond extreme heat stress (I) to Extreme heat stress, for heat stress beyond 41 °C. Although this reduction is significant, tree shade had limited impact under such extreme heat. The study found that trees on the left side were more effective when placed on one side, while the right side provided stronger cooling when trees were on both sides in both E-W and N-S streets. Additionally, during the El Niño period, the influence of street morphology on thermal comfort in '*Csa*' climates begins to reflect the conditions of '*BWh’* climates, with higher levels of heat stress. As climate change continues to intensify, these extreme heat conditions may become typical in the future.

## Introduction

The increasing heat risks associated with climate change, alongside irregular urbanization, underscore the significant challenges cities face in maintaining human comfort and public health. One such issue was the Urban Heat Island Intensity (UHII), which emerges in direct proportion to the increase in concrete surfaces, one of the most severe problems in urban areas (Pomerantz et al. 2003; Chen et al. 2019). The UHI effect negatively affected thermal comfort (Cao and Deng 2019; Xi et al. 2021; Ren et al. 2022), a crucial factor for evaluating local heat risk (ASHRAE 2017), often assessed using the Physiologically Equivalent Temperature (PET), a widely used index within Munich Energy Balance Model for Individuals (MEMI) (Höppe 1999; de Freitas and Grigorieva 2015; Chen and Matzarakis 2018; Staiger et al. 2019). The UHI effect leads to physical and psychological health problems (McKenzie 2009; Tomlinson et al. 2011; Nouri 2013; Ebi et al. 2021), highlighting the importance of accurately assessing microclimatic parameters. These parameters can be measured using in-situ methods (Qaid et al. 2016; Kim and Brown 2022, 2023; Kim et al. 2022; Melas et al. 2023), or meteorological station (Shevchenko et al. 2019; Pattisinai and Widayanti 2021; Yilmaz et al. 2021) and measurements from the meteorological station comparison of both datasets, with a limited number of studies conducted (Ali-Toudert and Mayer 2007; Szer et al. 2022; Haeri et al. 2023). Despite the advantages of in-situ measurements in capturing microclimatic variations with high spatial and temporal resolution, several limitations have been identified in previous studies, particularly related to equipment sensitivity, sensor calibration, and the influence of sensor positioning. Each environmental parameter—such as air temperature, relative humidity and wind speed and radiation fluxes —requires specific calibration procedures and accuracy thresholds to ensure valid and comparable results. These procedures need to be carefully undertaken to not just ensure effective estimations of human EBM dynamics and biometeorological processes, but moreover, to ensure that the portable instruments are properly utilized for such purposes, including with regards to radiation (Matzarakis et al. 2010) and wind impacts on humans (Kuttler 2000). More concretely, when focusing upon thermal human comfort studies, the use of meteorological stations enable comparisons to be undertaken with ‘in-loci’ locations of the public realm, including ascertaining complex dynamic variables such as radiation flux dynamics and wind patterns. Such methodologies present different technical challenges to those of a more hygrometric nature, which while meaningful within respective studies, carry different limitations in terms of understanding the impacts upon human thermo-physiological risk factors (Charalampopoulos and Santos Nouri 2019). For this reason, their methodical placement within specific locations and equipment calibration must also always ensure the correct evaluation of the versatile nature of the urban microclimate upon humans as described by (Höppe 1999). These requirements have been consistently addressed within later literature as well, along with the measurement protocols which have been adopted and validated by several studies (Cui et al. 2021; Sharma et al. 2022; Deng et al. 2023; Li et al. 2023). Understanding these differences is crucial for accurate heat risk assessment and effective local adaptation measures. It is particularly important to conduct studies during periods of temperature increases, especially during the El Niño phenomenon, which has been declared by the World Meteorological Organization (WMO) for the 2023–2024 period. El Niño, a periodic climatic phenomenon driven by changing wind patterns and warmer sea surface temperatures near the equator in the Pacific (McCreary 1976), increases minimum temperatures (Karabörk et al. 2005), and leads to high thermal stress during this period (Ramirez-Beltran et al. 2017; Roffe et al. 2023). Bottom-up methods were needed to understand the local heat risk factors upon humans on how local adaptation measures could be concretely introduced (Höppe 1984; Nouri 2013). Therefore, it is important to develop a comparative approach based on climate classifications defined on a regional scale, taking into account factors such as radiation and wind that represent in-situ conditions.

Thermal comfort was influenced by urban morphology: the Height-to-Width (H/W) ratio and Sky View Factor (SVF) which affect wind flow (Yang et al. 2013) and are shaped by both built structures and vegetation (Bourbia and Boucheriba 2010; Sharmin et al. 2019; Zaki et al. 2020a; Al Haddid and Al-Obaidi 2022; Zhu et al. 2022). Thermal comfort varies with the urban fabric, as H/W ratio and street orientation emerge as key factors defining urbanization(Ali-Toudert and Mayer 2006; Lai et al. 2019; Xiong et al. 2022; Cárdenas-Jirón et al. 2023; Nouri et al. 2023b). The aspect ratio influenced the thermal comfort under different climatic conditions where 1.5 H/W ratio mostly defined as a threshold value (Ketterer and Matzarakis 2014; Rodríguez Algeciras et al. 2016; Karimimoshaver and Shahrak 2022). In addition street orientation also play a significant role in thermal comfort, where East–West (E-W) orientations resulted in the worst thermal comfort conditions (Ali-Toudert and Mayer 2006; Taleghani et al. 2015; Achour-Younsi and Kharrat 2016; Jamei et al. 2016; Deng and Wong 2020). On the other hand, North–South (N-S) orientations provided the highest thermal comfort conditions(Ali-Toudert 2005; Srivanit and Jareemit 2020; Acero et al. 2021). However, studies on which lateral side of the street is more comfortable are limited (Deng and Wong 2020; Nasrollahi et al. 2021). According to findings of several studies, ideal orientation for thermal comfort may vary according to proximity to the equator, climate classification, micrometeorological and in-situ conditions, even if the references are all from the Northern Hemisphere.

The studies have highlighted that the cooling effect of trees varies with street orientation and aspect ratio, significantly influencing thermal comfort (Zaki et al. 2020b).The role of vegetation in mitigating the UHI effect and enhancing thermal comfort depends on tree placement, canopy coverage, species, and their transpiration properties, which effectively reduce air temperatures (De Abreu-Harbich et al. 2015; Morakinyo et al. 2016; Skelhorn et al. 2016; Lin and Tsai 2017; Sanusi et al. 2017; Rahman et al. 2020; Krayenhoff et al. 2021). Thermal comfort of streets with and without trees were previously discussed in several studies (De Abreu-Harbich et al. 2015; Lee and Mayer 2016; Matzarakis and Fröhlich 2017; Nouri et al. 2018c; Zheng et al. 2018b; Battisti 2020; Labdaoui et al. 2021; Necira et al. 2024). In a study, it was observed that the canopy transmissivity of different tree species significantly affects people's thermal comfort, depending on the variation in radiation (short and long wave) received under the trees (Brown and Gillespie 1990). However, there remains a gap in the literature regarding the specific effects of different tree species and canopy densities on thermal comfort, particularly in relation to their spatial arrangement along streets (Aboelata and Sodoudi 2020; Xiao et al. 2024). Studies addressing how different tree planting configurations—such as placement on one side or both sides of the street—affect thermal comfort, are limited (Darbani et al. 2023). These factors can significantly influence cooling efficiency and the extent to which urban heat stress is mitigated. Although the thermal comfort differences between streets with and without trees had been extensively researched, a better understanding is needed of how the introduction of tree species to streets affects thermal comfort depending on canopy transmissivity. Therefore, influence of tree species with different canopy densities on radiation and thermal comfort in urban streets was investigated in this study.

Within the urban context of Istanbul, research on thermal comfort mostly focuses on mosques, campuses, and squares (Göçer et al. 2018; Rad and Afzali 2021; Atmaca and Gedik 2023), but the research on street morphology is limited. In light of future climate scenarios that are indicating increased heat risk in Istanbul (Demircan et al. 2017; IBB 2021), it is crucial to conduct further investigation to develop effective heat risk mitigation measures on public open spaces, and especially streets where people frequently spend time. Therefore, this study focused on analyzing worst-case scenarios concerning heat vulnerability, with particular attention to the implications for public health on the streets and the broader effects on urban energy balance. Within the urban context of Istanbul, this study aimed to evaluate the thermal comfort conditions during the El Niño phenomenon in July 2023 by examining the role of vegetation on improving thermal comfort across two different street orientations (E-W, N-S) by analyzing data obtained from the field measurements and the nearest meteorological station were comparatively analyzed. In densely built urban areas like Istanbul, where adding trees to the urban fabric could be challenging, determining the most suitable tree species for each side of the street was crucial for local governments in their efforts to enhance thermal comfort, especially considering the increasing future risks related to climate change. These findings were intended to enrich the literature by providing insights into thermal comfort conditions during the El Niño period according to the Köppen–Geiger (KG) classification for Istanbul and by drawing comparisons with the studies conducted with other KG classification (Peel et al. 2007).

## Methodology

### Study area

**Fig. 1:**
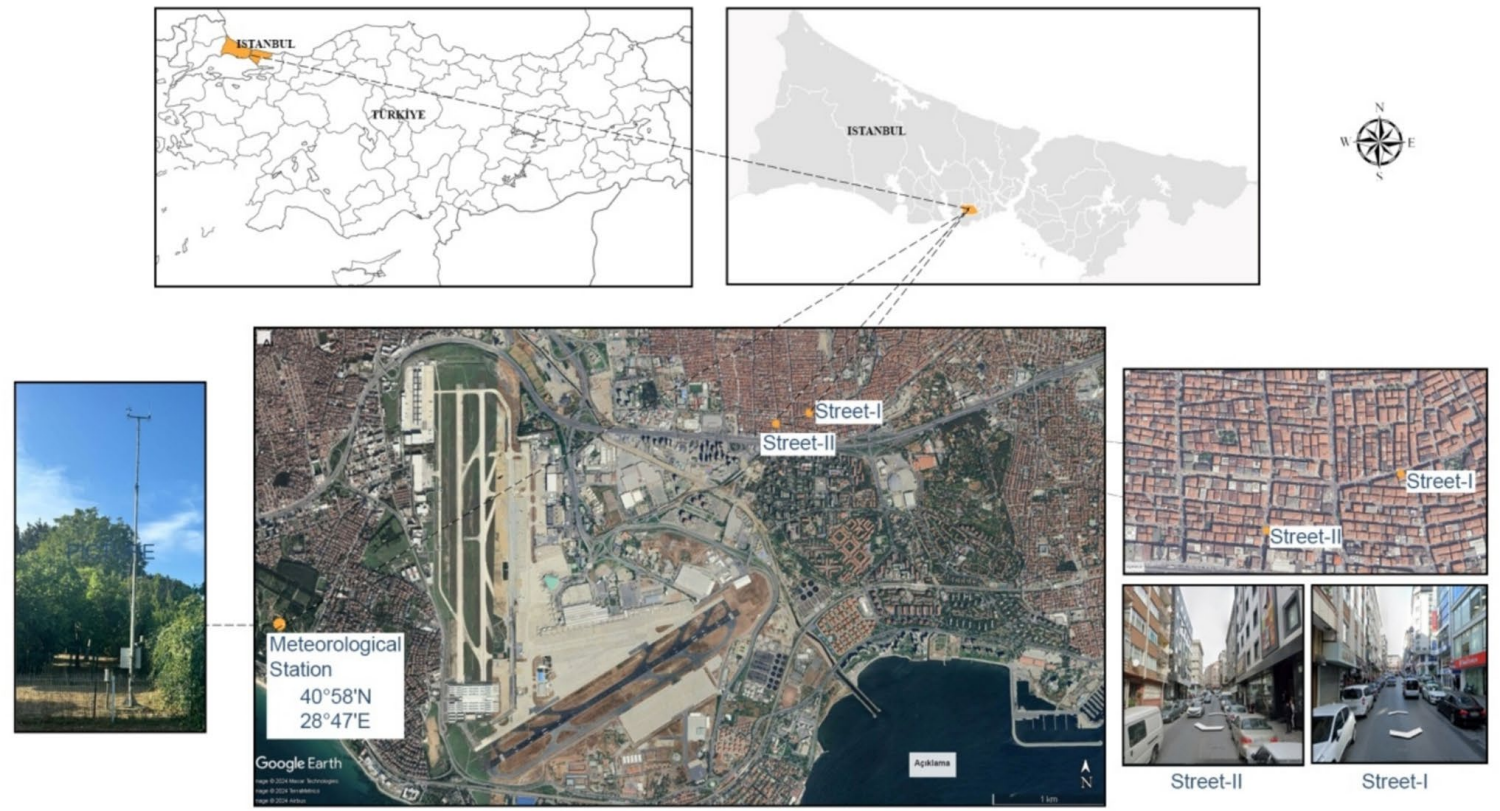
Location of the streets where measurements were taken

As a Türkiye's most populous city with around 15 million residents Istanbul spanning 5,461 km^2^, features a diverse climate (approximately 41° N latitude, 29° E longitude, with altitudes ranging from 10 to 537 m). The climate in Istanbul varies in the north–south direction where southern part experiences Mediterranean climate ('*Csa*'according to the KG), transitioning to a cooler"Black Sea Climate"in the north, influenced by sea and land air masses, as noted by Ezber et al. (2007). The general average temperature in July is between a minimum of 16 °C and a maximum of 30 °C. During years impacted by the El Niño phenomenon (Chen et al. 2016; Hayasaka and Sepriando 2018), the daily minimum (min) and maximum (max) temperature averages for July, recorded between 08:00 and 18:00 since 2009 by the Meteorology Station (MS), were presented in Appendix- 1. The study area is determined from the highest land surface temperature on a day of extreme heat events in Istanbul (Fig. 1).

### Field measurements and data collection

**Fig. 2:**
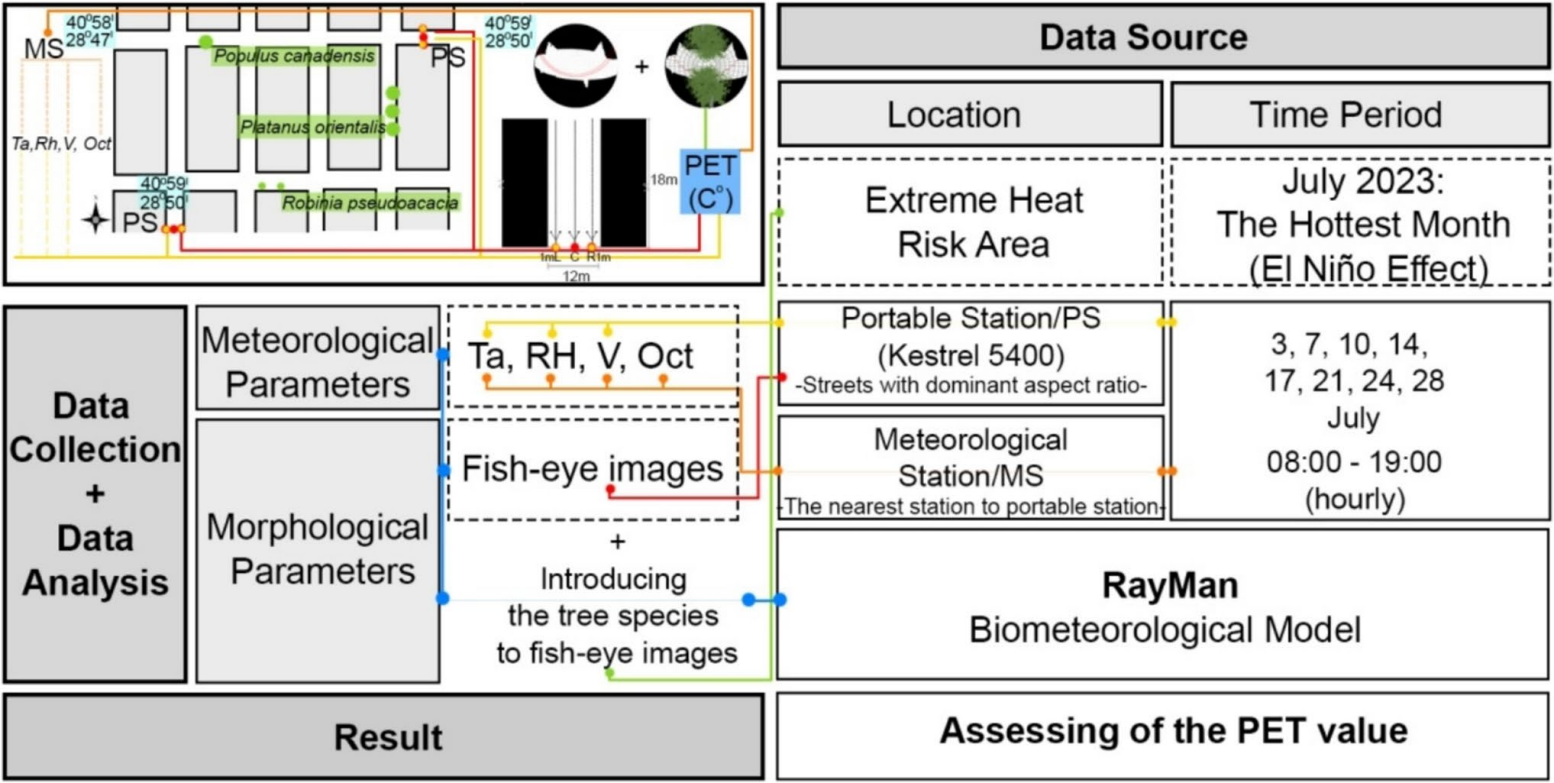
Research Methodology Framework Diagram

A method illustrated in Fig. 2 was developed to identify local risk factors in areas subject to “Extreme Heat Risk”, particularly on the hottest conditions were experiencing the hottest conditions. Extreme Heat Risk was determined using hourly temperature data from 1991–2021, based on two event types: Heat Wave Events(HWE) and Very Hot Days(VHD) (Zhang et al. 2005). HWE was defined as six or more consecutive days with maximum temperatures exceeding the 95 th percentile, while VHD used the 90 th percentile threshold (Smith et al. 2013; Piticar et al. 2019; Nouri et al. 2022). On August 5, 2021, an EHE day was identified, and the area with the highest land surface temperature was analyzed. In-situ measurements conducted in this area during the hottest month of 2023 enabled the evaluation of risk factors through systematic data collection and statistical analysis of meteorological and morphological parameters based on the identified extreme conditions (Fig. 2). In the first stage consist of comparison thermal comfort values derived from these two sets of meteorological data (portable station (PS) and meteorological station (MS)). Subsequently, the study evaluated the influence of in-situ meteorological and morphological parameters on thermal comfort for the E-W and N-S street orientations. In the second stage, the study investigated how thermal comfort changed across different street positions when trees from fisheye images of tree-lined streets were integrated into fisheye images of treeless streets.

#### Measurement configuration and meteorological parameters

**Fig. 3:**
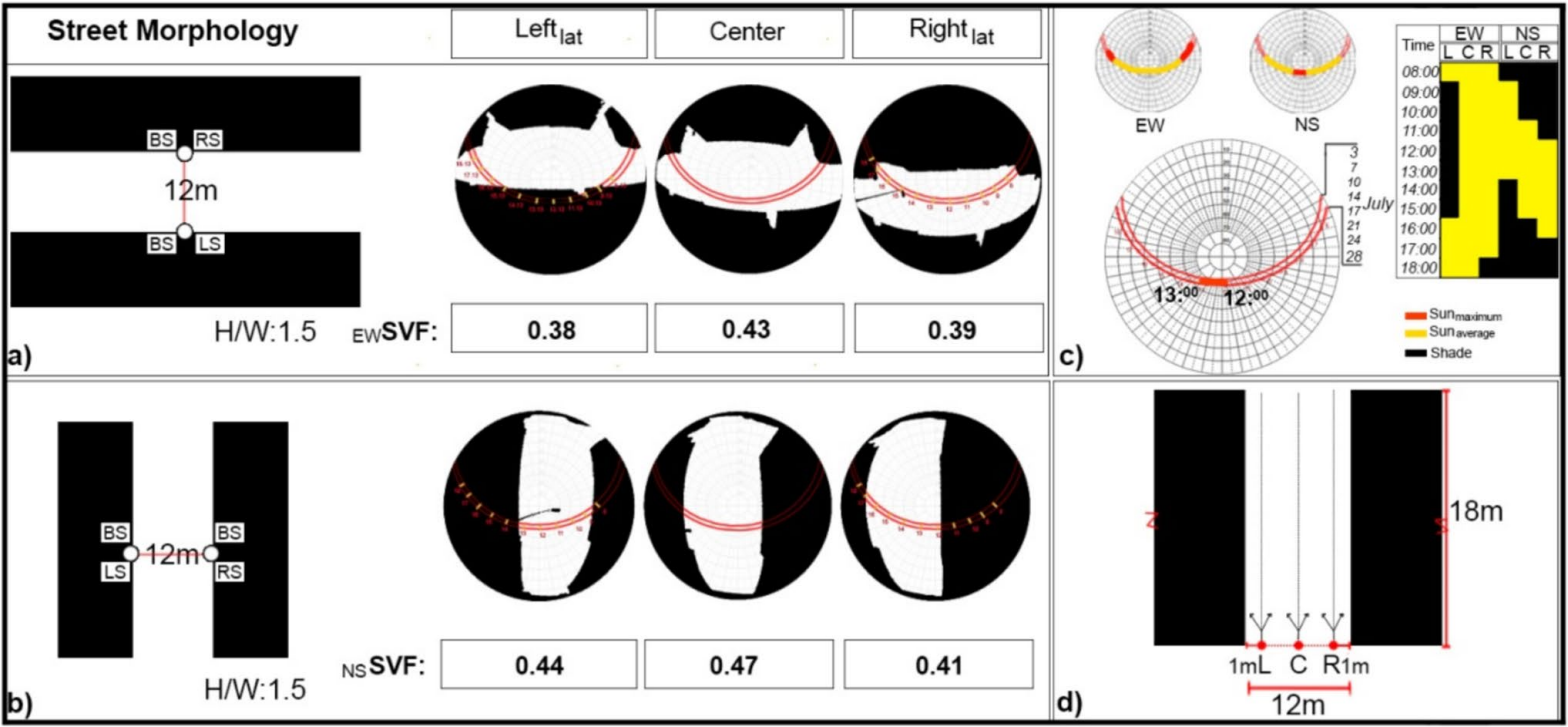
Morphological characteristics of the streets

Field measurements were conducted on a regular basis—specifically every Monday and Friday throughout July 2023—in order to ensure consistency in data collection and to enable generalizations about thermal comfort during the hottest month of the year (Appendix 2). Additionally, these streets represent a worst-case scenario, as they exhibited the highest land surface temperatures during extreme heat events. The specific location where the study was conducted lies at 40.59° N latitude, 28.50° E longitude, with an altitude of 20 m. Two streets with E-W/N-S orientations, were identified which demonstrates the general characteristic of the common typo-morphology of the study area. In accordance with a human-centered approach, meteorological measurements of air temperature (Ta), wind speed (V), and relative humidity (RH) were conducted at pedestrian level and compared with data from a nearby MS (with cloud cover (Octas) provided additionally), enabling a comprehensive comparison between the two datasets. Pedestrian level measurements performed from the PS, the measurement process included 13-min intervals on both the lateral points, known as Left Lateral (Left_Lat_) and Right Lateral (Right_Lat_), situated 1 m away from the respective façades to represent the'sidewalk area', the E-W (Street-I) and N-S (Street-II) oriented streets, starting from 08:00 and repeating every hour until 19:00 (Fig. 3) similar to the previous studies (Nouri et al. 2017, 2023a; Rodríguez-Algeciras et al. 2018). Measurements were carried out using the KESTREL 5400 local measuring device, which was mounted on a tripod set at a height of 1.1 m above the ground and collected data at a 1-min resolution. Each measurement parameter requires validation and thresholds (Appendix 2), an approach widely employed and validated by scholars in the field (Cui et al. 2021; Sharma et al. 2022; Deng et al. 2023; Li et al. 2023).

#### Morphological characteristics

Morphological parameters were obtained from two different streets where meteorological measurements were conducted using a PS, as well as from the specific points where these measurements were taken: E-W and N-S. The streets were the predominant aspect ratio for the area, with a width of 12 m and a building height of 18 m. To obtain fish-eye images, a fish-eye lens was used at approximately 1.5 m above the ground. Photographs were taken from the Right_lat_, center, Leftl_at_, of both streets. SVF values were obtained from fish-eye photographs (Fig. 3).

#### Selection of the tree species

The most common trees found in the nearby area were identified as *Platanus orientalis., Populus canadensis* and *Robinia pseudoacacia.* In addition to the diversity of tree species, the morphological conditions of the streets have developed differently from their natural environment*.* The characteristics and the growth of the selected tree species were examined (Appendix 3). Fish-eye photographs were also taken from the streets where these trees were located, at a height of 1.5 m and subsequently adapted based on Adobe Photoshop to the streets where measurements were conducted. The adaptation of the photographs was carried out in a manner that allows for the clear identification of leaf characteristics. Therefore, the differences in the radiation emitted by tree species, based on their leaf characteristics, could be understood, as noted by Brown and Gilissp (1990). The adaptation was done based on a'what if'approach where the street had trees on the left, both, or right side, as shown in Fig. 3. Appendix 4A demonstrates how *Platanus orientalis* affects SVF and sun path under different street orientations, with limited canopy diameter restricting shading and SVF impact. LS and RS scenarios resulted in increased sun exposure. Appendix 4B illustrated how *Populus canadensis*, with its larger canopy diameter, influences SVF and sun path under various street orientations, as well as its effect on sun exposure duration. Appendix 4C showed how *Robinia pseudoacacia*, with its smaller, paired leaflets, affected SVF and sun path under different street orientations. Its morphology allowed more sunlight penetration, resulting in longer sun exposure durations between 08:00 and 18:00 compared to other species.

### Analysis of human thermo-physiological thresholds

Thermal comfort in urban public spaces was assessed using various indices (van Hoof 2008; Deb and Ramachandraiah 2010; Staiger et al. 2012) including UTCI, SET*, and PET (Xi et al. 2012; Staiger et al. 2019; Potchter et al. 2022). PET index, derived from the MEMI model (Höppe 1984, 1999a) and accounting for the human body's entire heat budget to measure comfort in °C, was applied in this study. Upon processing the PET values, the temperatures obtained were classified into specific physiological stress levels indicated in Table 1, utilizing the comparative table proposed by Matzarakis and Mayer (Matzarakis and Mayer 1996). The PET classification system was expanded to more accurately assess thermal stress, particularly beyond the original ‘extreme heat stress’. To address temperatures exceeding 41 °C, additional categories were introduced, based on increments of 5 °C. This extension provided a framework for understanding the impact of severe heat on the human biometeorological system, while also aligning with existing research on the calibration and distribution of thermophysiological indices (Hwang and Lin 2007; Lin and Matzarakis 2008; Nouri et al. 2018 d, a, 2021). By integrating new heat stress categories into the existing classification framework, this also extended previous results of extreme classification scenarios (Table 1).

**Table 1:** Description of physiological stress based on PET

PET (^o^C)	Grade of physical stress	Stress Level Abbreviation
18 to 23	No thermal stress	(NTS)
23 to 29	Slight heat stress	(HS1)
29 to 35	Moderate heat stress	(HS2)
35 to 41	Strong heat stress	(HS3)
41 to 46	Extreme heat stress	(HS4)
46 to 51	Beyond extreme heat stress I	(HS5)
> 51	Beyond extreme heat stress II	(HS6)

^a^Ranges of the PET for different grades of Physiological Stress (PS) on human beings. These values are calculated based on standard conditions, including a heat transfer resistance of clothing set to 0.9 clo and an internal heat production of 80 W, as described by Matzarakis and Mayer in 1996 (Matzarakis and Mayer 1996)

The RayMan biometeorological model calculates long-wave and short-wave radiation flux densities, thermal comfort indices such as PET for a single location, based on meteorological inputs, morphologic and personal parameters (Matzarakis et al. 2007, 2010). The study incorporated V and Octas into the PET calculations through the RayMan model, which adjusted for wind patterns and radiation fluxes (Matzarakis et al. 2007, 2010, 2016, 2021; Matzarakis and Fröhlich 2018). For morphological parameters, building modeling that provided the fish-eye photographs, raw and tree-added fish-eye photographs were utilized. Meteorological parameters derived from PS and MS. In addressing the dynamics of V within the urban canopy and along street corridors, a significant deviation from MS is noted (Oliveira et al. 2011). This phenomenon necessitated a recalibration of V to more accurately measure the wind velocities impacting pedestrians at the ground level. Therefore, MS data was modified to reflect conditions at a pedestrian's height of 1.1 m. This recalibration, utilizing a formula validated in the literature (Kuttler 2000), with α defined by urban texture, serves to align meteorological insights with the pedestrian experience.

$$V1.1=Vh\times {\left(\frac{1.1}{h}\right)}^{\alpha }\alpha =0.12\times {z}_{0}+0.18$$

α is an empirical exponent, depending upon urban surface roughness, z_0_ is the corresponding roughness length. For the study area, depending on the roughness of the urban fabric the following calibrations to the formula:

z0 = 1.8 and α = 0.4

### Statistical analysis

Within the scope of the study, inferential statistical analysis was performed using IBM SPSS Statistics to reveal the difference in thermal comfort across various street orientations and lateral areas. The significance threshold for statistical analysis was set at 0.05 (confidence interval = 95%). Initially, an ANOVA t-test analysis was performed to compare the difference between PET values and physiological stress levels in N-S– E-W oriented streets based on varying meteorological and morphological parameter inputs. This comparison included:

- Meteorogical parameters obtained from MS and PS,
- PET values obtained from field measurements along with fish-eye photographs in terms of the street orientation.

Secondly, the change in thermal comfort when different tree species (*Platanus orientalis, Populus canadensis, Robinia pseudoacacia*) were added to fish-eye photographs was analyzed. Three assessments were undertaken to understand the implications of the following; (i) What if the three different tree species were placed on the right side (RS) of the street? (ii) What if the three different tree species were placed on both sides (BS) of the street? (iii) What if the three different tree species were placed on the left side (LS) of the street?

Based on these questions, an ANOVA test was conducted to compare the differences in PET values of E-W/N-S oriented streets according to the presence of trees in different lateral areas of the street and among different tree types. To identify differences between the results, these were further analysed using Tukey's test for multiple comparisons, which was performed to specifically assess differences between group means. A comparison of sun and shade conditions based on PET differences between streets with and without trees was conducted. As the data did not meet the assumption of normality, the Mann–Whitney test was used for the analysis.

## Results

### Assessment of thermal comfort based on meteorological variables and morphological characteristics

A comparison of meteorological data between the PS and MS showed significant differences in Ta and RH (p = 0.00). Specifically, it was found that Ta was 1.7 °C higher in PS compared to MS, while RH was 10% higher in MS compared to PS. Additionally, no statistically significant difference was found between the two stations in terms of V (Appendix 5). Analysis of the data from the meteorological station indicates that the most severe conditions occurred on July 7, 14, and 21, with HS5 conditions notably persisting from 10:00 to 17:00 on July 14. However, the PET values derived from the meteorological station, due to their lack of representation of specific street locations, did not permit detailed spatial inferences (Appendix 6A). A statistically significant difference was found between E-W and N-S, with PET values being higher in E-W(Mean: 40.6) than in N-S (Mean:39.5) based on the in-situ measurements (p = 0.00). Moreover, the E-W—Left_lat_ consistently exhibited at least HS5 conditions on all measurement days, while the most severe conditions were observed in the E-W Center and Right_lat_. On July 7, 14, and 21, the extreme conditions lasted for up to 6–7 h (Appendix 6B).

**Fig. 4:**
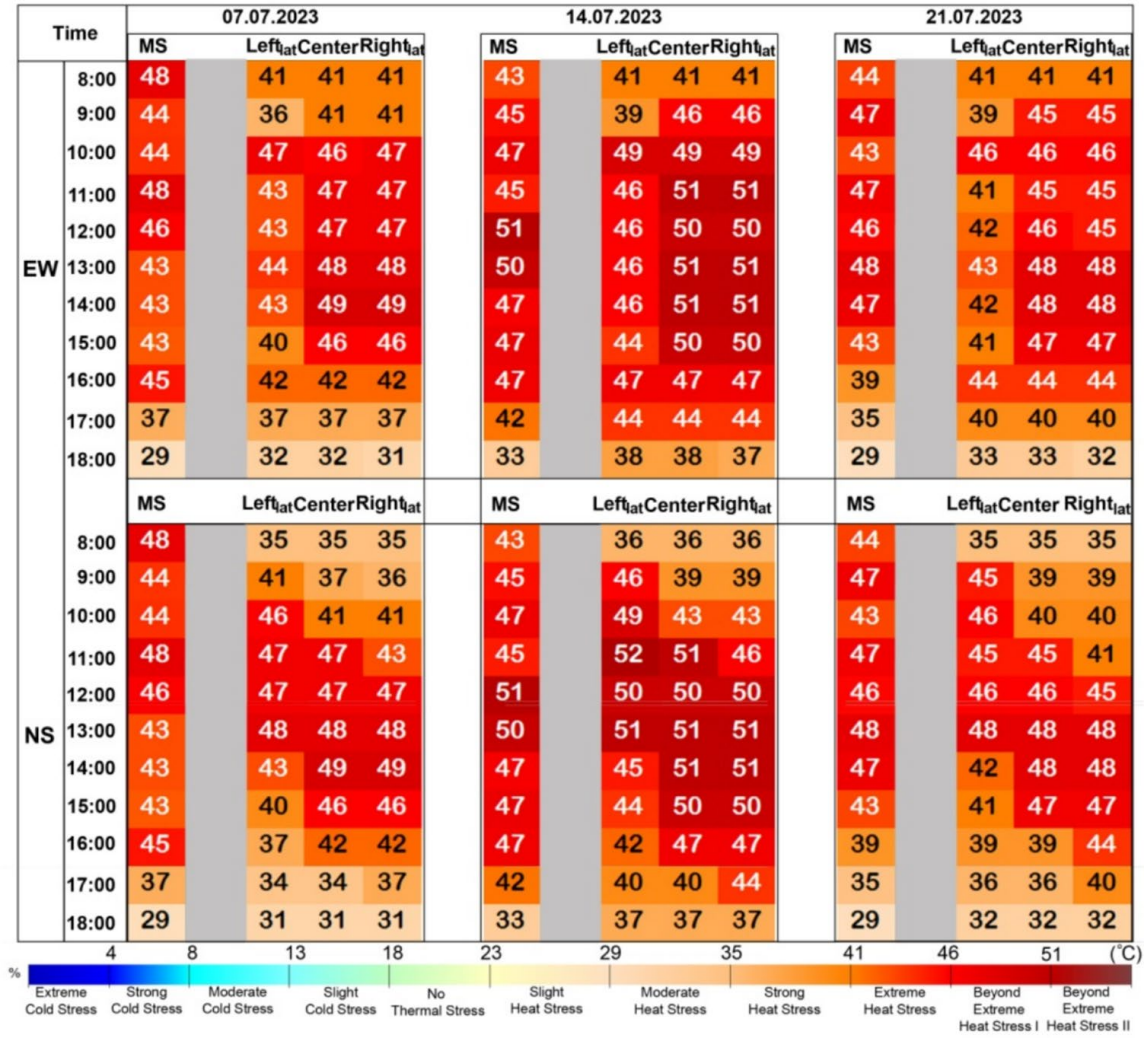
Hourly PET variations for E-W and N-S street orientations at Leftlat, Center, and Rightlat of PS and MS on selected 7, 14, 21 July days without the presence of trees, and serving as a base analysis for the subsequent scenarios which hosted specific vegetation layout typologies

On the most extreme conditions, PET values obtained from MS and PS were compared according to time intervals. Extreme heat stress was observed at 8:00 in the MS, while strong heat stress occurred in both E-W and N-S streets at the same time. By 9:00, the MS reached extreme heat stress, with similar conditions observed in the E-W at Center and Righ_lat_, and in the N-S at Left_lat_ on July 14 and 21. Between 10:00 and 16:00, beyond extreme heat stress persisted in the Center and Righ_lat_ across all days, while Left_lat_ varied, reaching the extreme heat stress threshold on July 14 in the PS. In the N-S street, PET peaks were consistent across all days, with beyond extreme heat stress observed in Left_lat_ from 9:00 to 13:00, in Center from 11:00 to 16:00, and in Righ_lat_ from 12:00 to 16:00. On July 14, nearly all locations approached the HS5 threshold, with PET values peaking at 50–51 °C between 11:00 and 13:00.In the MS, there were inconsistencies in days and times compared to specific locations. PET values in the E-W street showed that Left_lat_ was generally lower than the MS, while Center and Righ_lat_ were higher. In the N-S street, values were generally lower than the MS, with limited similarity on July 14 and 21 between 12:00 and 13:00. Differences in PET values across E-W and N-S streets corresponded to variations in sun exposure duration (Fig. 4).

### Assessment of thermal comfort based on the presence of different tree species in the lateral areas of the street

The comparative analysis of tree placement within different street orientations—specifically their positions on the LS, BS, and RS of streets—revealed significant thermal comfort implications. The effect of trees positioned on the LS and RS on thermal comfort showed no significant differences in reducing PET values between tree species in both E-W and N-S orientations. However, trees positioned on BS demonstrated significant differences in their impact on reducing PET values among tree species, with notable variations in the E-W (p = 0.015) and N-S (p = 0.017) orientations. However, the PET values at these points varied depending on the positioning of the trees in different lateral areas of the streets. Notably, in the center of EW, configurations with trees located on the LS or on the BS resulted in lower PET measurements compared to scenarios where trees were positioned on the RS. Similarly, for the Right_lat_ of these streets, arrangements with trees on the BS or on the RS yielded lower PET values than configurations with trees only on the LS. The Left_lat_ of N-S exhibited lower PET values when trees were present either on the LS or on the BS, in contrast to conditions with trees only on the RS. In the center of NS, trees positioned on BS facilitated lower PET measurements compared to scenarios where trees were found only on the RS. For the Right_lat_ of the N-S, the presence of trees on the BS or on the RS resulted in lower PET values compared to cases with trees exclusively on the LS. Below, the differences among tree species according to their presence in different sides of the street, and the differences in the Left_lat_, center and Righ_lat_ of the streets are presented.

#### Impact of different trees positioned on the E-W oriented street on thermal comfort

On July 14, under the worst conditions, when trees were located only on the LS and on BS, in the E-W oriented street in the Left_lat_ area, all tree species exhibited a shift from HS5 to HS4 at 10:00. However, by 16:00, only *Populus canadensis* demonstrated this shift from HS5 to HS4.In the center of the street, *Platanus orientalis* was effective in moving from HS4 to HS3 at 09:00, and from HS5 to HS4 at 10:00. Between 11:00 and 14:00, all tree species contributed to the shift from HS5 to HS4, while from 12:00 to 13:00, only *Platanus orientalis* and *Populus canadensis* were effective in this change. When trees were located only on the right side, no changes were observed in PET values Left_lat_ or Center of the E-W oriented street. When trees were located only on the left side, in the Right_lat_ area, there were no observed changes in PET values for any of the tree species. When trees were located on both sides, in the Right_lat_, at 09:00, *Platanus orientalis* shifted from HS4 to HS3, while on the right side, heat stress shifted from HS5 to HS4. When trees were located on both sides, or only on the right side, between 10:00 and 13:00, and at 15:00, both *Platanus orientalis* and *Populus canadensis* reduced the heat stress from HS5 to HS4. At 14:00, all tree species were effective in this change.

**Fig. 5:**
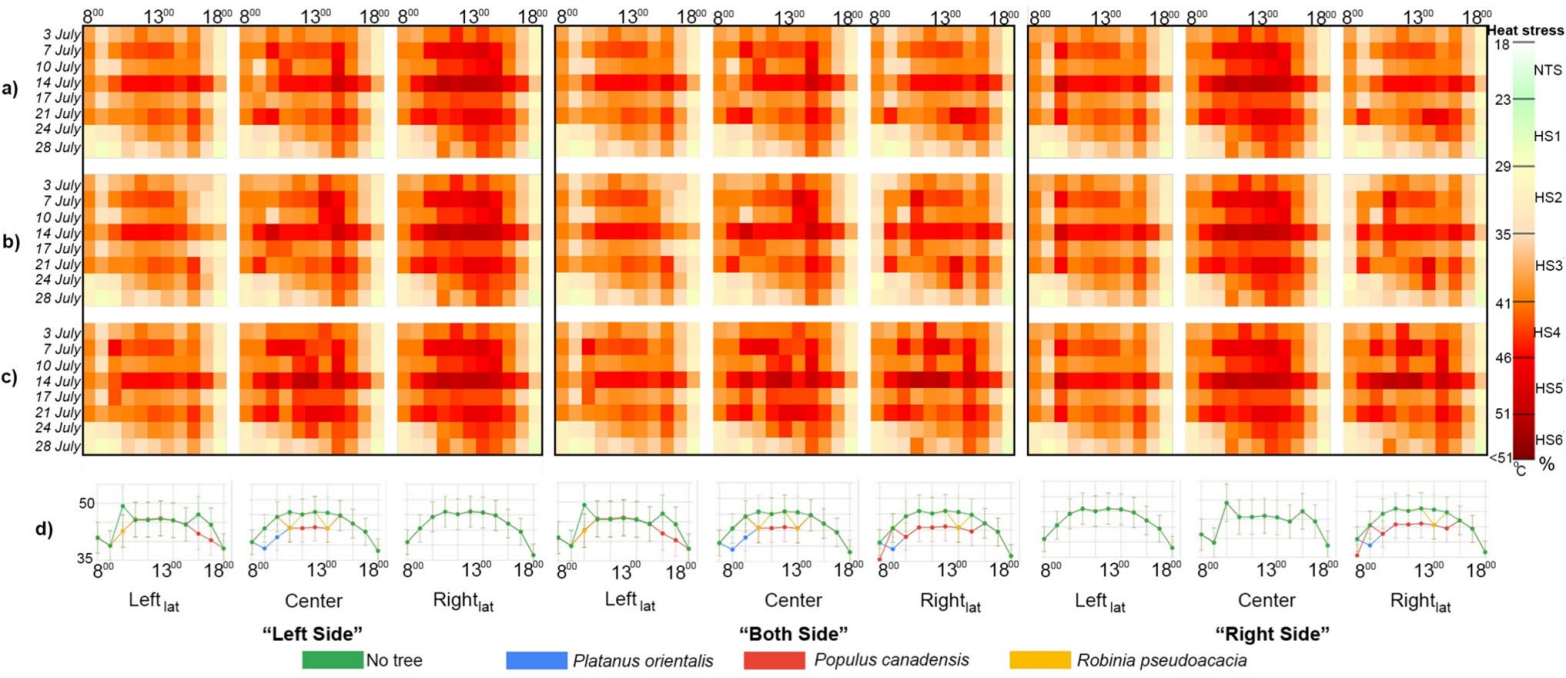
Hourly PET variations for E-W street orientations at Left_lat_, Center, and Right_lat_ on selected July days, with trees planted at different positions—Left Side (LS), Both Sides (BS), and Right Side (RS). Subfigures illustrate: **a**) *Platanus orientalis*, **b**) *Populus canadensis*, **c**) *Robinia pseudoacacia*, and **d**) the hottest day (14 th July), comparing PET conditions with trees planted on LS, BS, RS, and the treeless scenario

When trees were located only on the LS, *Platanus orientalis* led to a 4–6 °C temperature reduction in the Left_lat_ at 10:00 (across all days) and a 3–7 °C reduction in the center (10:00–14:00). When trees were added on BS, *Platanus orientalis* caused a 4–6 °C drop at the Left_lat_ (10:00), 3–7 °C in the center (11:00–14:00), and 4–5 °C at the Right_lat_ (09:00–15:00). Similarly, *Populus canadensis* reduced temperatures by 3–5 °C in the Left_lat_ (10:00–17:00), 3–5 °C in the center (10:00–14:00), and 5–6 °C in the Right_lat_ (08:00–15:00). *Robinia pseudoacacia* had no effect on the Left_lat_ but decreased temperatures by 4–5 °C in the center (10:00–14:00) and the Right_lat_ (11:00–14:00). When trees were located only on the RS, *Platanus orientalis* caused a 4–5 °C reduction in the Right_lat_ (09:00–15:00), *Populus canadensis* by 4–5 °C (08:00–15:00), and *Robinia pseudoacacia* by 3–5 °C (11:00–14:00) Fig 5.

#### Impact of different trees positioned on the N-S oriented street on thermal comfort

In the N-S oriented street, changes were less similar to those observed in the E-W street. When trees were located only on the LS, in the Left_lat_, *Robinia pseudoacacia* was effective in the shift from HS5 to HS4 only at 10:00, whereas *Platanus orientalis* and *Populus canadensis* contributed to this shift between 10:00 and 13:00. No changes were observed after 14:00. In the Centre area, *Populus canadensis* showed a shift from HS5 to HS4 between 13:00 and 14:00, and from 15:00 to 16:00 all tree species showed a shift from HS5 to HS4. In the Right_lat_ area, *Populus canadensis* shifted from HS5 to HS4 at 15:00 and further to HS3 at 17:00. When trees were located on BS, in the Left_lat_ area, *Platanus orientalis* was effective in the shift from HS4 to HS3 at 09:00. At 10:00, all tree species were involved, and between 11:00 and 13:00, only *Platanus orientalis* and *Populus canadensis* contributed to the reduction from HS5 to HS4. In the Center area, at 11:00, both *Populus canadensis* and *Platanus orientalis* were effective in this shift, while between 12:00 and 14:00, only *Populus canadensis* was involved, and between 15:00 and 16:00, all tree species contributed to the reduction from HS5 to HS4. In the Right_lat_ area, *Platanus orientalis* and *Populus canadensis* contributed to this shift at 11:00, 14:00, and 16:00, while all tree species were involved between 12:00 and 13:00. When trees were located only on right side, shift from HS4 to HS3 was observed in the Left_lat_ area at 09:00. In the center of the street, at 11:00, only *Populus canadensis* exhibited a shift from HS5 to HS4, while at 12:00, both *Populus canadensis* and *Platanus orientalis* demonstrated this change. In the Right_lat_ area, shifts from HS5 to HS4 were observed at 12:00, 15:00, and 17:00 for both *Populus canadensis* and *Platanus orientalis*. Additionally, between 13:00 and 14:00, all tree species contributed to the shift from HS5 to HS4, while at 16:00, *Platanus orientalis* was effective in this change.

**Fig. 6:**
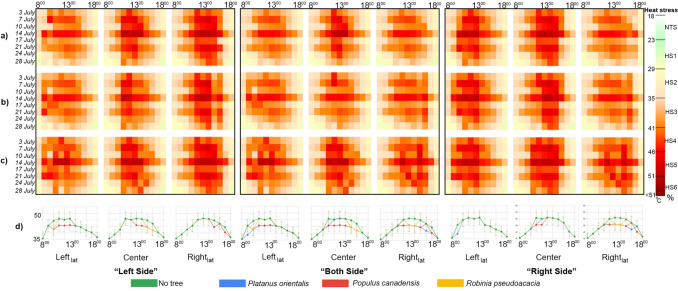
Hourly PET variations for N-S street orientations at Left_la_t, Center, and Right_lat_ on selected July days, with trees planted at different positions—Left Side (LS), Both Sides (BS), and Right Side (RS). Subfigures illustrate: **a**) *Platanus orientalis*, **b**) *Populus canadensis*, **c**) *Robinia pseudoacacia*, and **d**) the hottest day (14 th July), comparing PET conditions with trees planted on LS, BS, RS, and the treeless scenario

When trees were located only on the LS, *Platanus orientalis* reduced PET by 3–7 °C in the Left_lat_ (9:00–13:00), 4–7 °C in the center (14:00–16:00), and 3–4 °C in the Right_lat_ (17:00). *Populus canadensis* showed similar trends but did not consistently reduce PET in the Left_lat_ across all days. *Robinia pseudoacacia* caused reductions of up to 7 °C in the center (15:00) and occasionally 3 °C in the Right_lat_ (17:00) and in the Left_lat_ (10:00–12:00, 14:00). When trees were located on the BS, *Platanus orientalis* caused decreases in the Left_lat_ (9:00–13:00), in the center (11:00, 14:00–16:00), and in the Right_lat_ (12:00–17:00). *Populus canadensis* showed reductions similar to *Platanus orientalis* in the Left_lat_ but lacked consistency across days. In the center, reductions occurred (11:00–16:00), and in the Right_lat_ (12:00–15:00, 17:00). *Robinia pseudoacacia* caused rare decreases in the Left_lat_ (10:00, 12:00), more consistent reductions in the center (14:00–16:00), and in the Right_lat_ (13:00–14:00). Reductions in PET typically ranged between 3–5 °C. When trees were located only on the RS, no reductions were observed in the Left_lat_ with *Populus canadensis* or *Robinia pseudoacacia*, but *Platanus orientalis* caused a 3–5 °C reduction (9:00–10:00). In the center, *Platanus orientalis* and *Populus canadensis* reduced PET by 2–5 °C (11:00), while *Robinia pseudoacacia* caused no change. On the Right_lat_, *Platanus orientalis* reduced PET by 3–5 °C (12:00–16:00), *Populus canadensis* by 3–6 °C (12:00–17:00), and *Robinia pseudoacacia* by 3–5 °C (12:00–14:00) (Fig. 6).

### Effectiveness of tree species in improving thermal comfort conditions

In the E-W orientation, *Platanus orientalis* and *Populus canadensis* significantly reduced PET, lowering thermal stress from HS5 to HS4 during morning and midday hours. Conversely, *Robinia pseudoacacia* showed irregular effects, with no consistent changes in the Right_lat_. In the N-S orientation, Platanus orientalis and *Populus canadensis* were particularly effective in the Left_lat_ and centre. On the day with the worst conditions, *Populus canadensis* was particularly effective in altering heat stress. Overall, trees with broad shading capacity proved more effective in areas with intense sun exposure but showed limited improvements in heat stress except for specific hours. It was observed that *Robinia pseudoacacia* did not have a similar effect due to its irregular leaf structure and other morphological characteristics. PET reductions were observed for all species, with *Platanus orientalis* showing the greatest decrease. The relationship between the shade effect and PET differences after the addition of trees was analysed. The change in Rightl_at_ in N-S for *Platanus orientalis*, Right_lat_ in E-W for *Populus canadensis* and centre in E-W and Right_lat_ in N-S for *Robinia pseudoacacia* was not statistically significant. All other changes were significant. Although it did not completely improve the heat stress conditions, it was noteworthy that it decreased the PET value during the shade period (Appendix 7A, 7B).

## Discussion

### The impact of meteorological and morphological parameters on thermal comfort

Meteorological and morphological parameter inputs have been contrasted with meteorological parameters derived from MS and PS. In PS, the Ta was found to exceed values from MS, while RH and V were higher in MS. This discrepancy between meteorological parameters aligns with the findings of Szer et al. (2022) (Szer et al. 2022). When assessing PET values, a notably distinct difference emerged on the left side of E-W oriented streets, with PS yielding higher PET values. PS provided more realistic data about the study area, enabling the examination of differences among morphological parameters using in-situ input variables. Assessments based solely on MS were found to be insufficient compared to the comprehensive evaluations facilitated by PS. These findings underscored the necessity of adopting a more holistic approach to urban microclimate analysis, particularly in complex environments such as street canyons and inner regions. By integrating PS data, urban planners and landscape designers can better understand microclimatic effects, develop climate resilience strategies, and inform sustainable urban design and municipal guidelines.

Between 9:00–10:00, thermal stress on the Left_lat_ of both E-W and N-S streets was higher compared to MS, with E-W streets experiencing greater heat stress due to prolonged sunlight exposure (Ketterer and Matzarakis 2014). Under H/W:1.5 conditions, using a 9-year July average as the meteorological parameter, HS3 was observed between 11:00–14:00 in E-W streets and HS2 between 11:00–17:00 in N-S streets (Cárdenas-Jirón et al. 2023). For H/W:2, HS4 was recorded between 12:00–16:00 in E-W streets and 10:00–12:00 in N-S streets (Sözen and Oral 2019), In contrast, with an H/W:1 ratio, HS3 persisted from 10:00–16:00 in E-W streets, while N-S streets experienced HS3 between 10:00–14:00 (Andreou 2013). The'*Cfa*'climate classification, which shares comparable summer temperatures with'*Csa*'(Nouri et al. 2018b), also exhibited extended heat stress in E-W streets. For H/W:2, HS4 occurred on the Left_lat_ of E-W streets between 9:00–16:00 and on the Right_lat_ between 7:00–9:00; in N-S streets, HS4 was observed on the Left_lat_ between 10:00–12:00 and on the Right_lat_ from 12:00–14:00 (Deng and Wong 2020). The findings were consistent with those from similar climate classifications regarding the longer durations of thermal stress conditions in E-W orientations. While other studies observed a shift from HS4/HS5 to HS3/HS2 conditions after 12:00 in N-S orientations, continuing until 15:00, in contrast to the reduced compatibility in N-S orientations within the'Csa'and'Cfa'type, research in the'*BWh*'climate classification reported HS4 lasting from 08:00 to 19:00 in both E-W and N-S orientations, with a July study noting HS4 occurring between 10:00 and 16:00 in E-W and between 11:00 and 14:00 in N-S orientations.(Ali-Toudert and Mayer 2006; Nasrollahi et al. 2021).

Thermal comfort conditions in the'*Csa*'climate type under the influence of El Niño showed similar higher levels of heat stress as those typically observed in the'*BWh*'climate type, which is characterised by higher annual air temperature and lower precipitation for both summer and winter periods. This study draws attention to the compatibility of these findings with hot desert climate results resulting from the El Niño effect. A positive correlation between the thermal comfort index UTCI and El Niño had been identified in previous research (Ullah et al. 2024). Studies based on future climate scenarios have shown that PET values could increase by an average of 7.5 °C. In this context, it was clear that the results obtained in the worst-case scenario could become typical in the future (Matzarakis and Amelung 2008; Nouri et al. 2023b). This highlights the need for local governments to implement both short-term strategies, such as heat warning systems and incorporating shading into street design, and long-term measures, such as heat risk mapping.

### The impact of tree species on improving thermal comfort

In this study, the influence of tree species on thermal comfort was assessed by integrating tree canopies into SVF calculations. *Robinia pseudoacacia* was identified as the least effective species, with an average PET reduction of only 1 °C between 08:00 and 18:00 over eight days. Similarly, a study investigating the cooling effects of *Robinia pseudoacacia* reported an average PET reduction of 1.6 °C (Rahman et al. 2020). It was found that the impact of *Populus canadensis* and *Platanus orientalis* on thermal comfort varies significantly depending on which side of the street they are planted. For instance, in the'*Cfa*'climate classification with a crown diameter of 8 m, trunk height of 5 m, and total height of 12 m, *Populus canadensis* planted on both sides of the street was analyzed for E-W and N-S oriented streets. Both orientations exhibited HS4 between 12:00 and 15:00 on both sides of the street. The cooling effect of the trees on the Right_Lat_ of the street occurred between 9:00 and 16:00, while on the Left_Lat_, it occurred between 8:00 and 16:00. In the N-S orientation, cooling on the Left_Lat_ of the street occurred from 09:00 to 12:00, and on the Right_Lat_ from 11:00 to 14:00 (Xiao et al. 2024). HS3 shifts for *Populus canadensis* were noted at different times on both the Left_lat_ and Right_lat_, which was attributed to variations in crown diameter and trunk height, significantly affecting shading and thermal exposure (Park et al. 2019; Lee et al. 2020; Gachkar et al. 2021). According to Xiao et al. 2024 the PET reductions for *Platanus orientalis* were the same as for *Populus canadensis* because both were adjusted to have the same crown diameter, trunk height, and tree height. *Platanus orientalis* was reported to be more effective in improving thermal stress during summer than other deciduous tree specied due to its canopy coverage (Zhang et al. 2022). However, in this study, the results differed due to variations in morphological parameters such as crown diameter, highlighting the important of tree species selection in urban planning. These morphological differences not only affected shading capacity but also emphasized the critical role that spatial arrangement plays in optimizing the effectiveness of trees in blocking sunlight at specific locations (Brown et al. 2015). In addition, in scenarios where trees can only be placed on one side, it was found that the left side leads to more effective PET results, even without manipulating the sun path with shade.

Numerous studies have demonstrated the cooling effect of trees in urban settings, with variations depending on climate and street orientation. In'*Csa*'climates, tree cooling effects were most pronounced during the hottest hours (Mandelmilch et al. 2020). In a study conducted in a'*Cfa*'climate, HS4 was observed between 10:00 and 17:00 without trees, and between 13:00 and 15:00 with trees (Zheng et al. 2018a). In a BHW climate, HS4 was observed between 10:00 and 17:00 in an EW-oriented street without trees, while in scenarios with 20% and 50% tree coverage, the stress level remained unchanged between 12:00 and 17:00. In the N-S direction, HS4 was observed between 11:00 and 16:00 without trees, but in both tree coverage scenarios, a decrease was provided, with only the 70% tree coverage scenario shifting to HS3 (Aboelata and Sodoudi 2020). This highlights how tree coverage can significantly mitigate heat stress, though its effectiveness depends on local microclimatic conditions and the spatial arrangement of trees. In this study, with trees on both sides, HS4/HS5 was observed on the Left_lat_ from 11:00–16:00, and on the Right_lat_ from 12:00–16:00. Similarly,'*BSk*'studies showed worse thermal conditions on the Right_lat_ in E-W streets and on the Left_lat_ in N-S streets, with stronger cooling effects on the Right_lat_ in both orientations (Ma et al. 2019; Darbani et al. 2023). Differences in cooling effects between Left_lat_ and Right_lat_ configurations correlated with sunlight exposure. While shading provided some improvement, these effects were insufficient to completely mitigate heat stress conditions. While factors such as street orientation and the side on which trees are planted are important, in some cases – particularly when levels of extreme heat stress are reached – even trees may be insufficient to effectively mitigate such extreme heat. Therefore, urban planning must incorporate not only tree planting but also more comprehensive and multilayered cooling strategies.

## Limitation

This study was conducted in an area with the'*Csa*'climate type, and the results may not be applicable to other climate types. Furthermore, the study only focused on certain tree species, including *Platanus orientalis*, *Populus canadensis* and *Robinia pseudoacacia*, suggesting that local preferences in different cities, existing tree species or ecological factors may alter the observed results. In addition, the variability in leaf transmittance due to the developmental characteristics of the trees can influence the radiation levels under the canopy. Another important factor influencing thermal comfort under the canopy is wind, which does not affect the canopy and lower branches in the same way. Future studies should further investigate the relationship between trees and wind dynamics. The study also did not consider the cooling effect of trees through evapotranspiration. Future research should consider evapotranspiration and examine the interaction between trees and surface vegetation, such as grass, to better assess cooling effects in different street settings.

## Conclusion

Consequently, although worst-case scenarios have already been selected in this study, these scenarios will increase as a result of climate change effects within the urban fabric. It is therefore anticipated that the selected worst-case scenarios will worsen as a result of the effects of climate change on the urban fabric. This emphasises the necessity for adaptation to both current and future conditions. With the intensifying impact of climate change, what are considered extremely hot conditions today may be perceived as a ‘typical’ or even a ‘cool’ summer day by the end of the century. Such a shift will directly impact human health and quality of life, highlighting the need for municipalities to adopt adaptation measures. Effective strategies in public spaces, including heat action plans and vulnerability maps, must consider diverse methodologies to address climate change impacts. For example, in EW-oriented streets – particularly Leftl_at_, higher levels of extreme heat stress (HS5) have been consistently observed, further emphasizing the importance of incorporating street orientation and microclimate analyses into urban planning and climate adaptation strategies. This study demonstrates that the effects of street orientation and morphology on thermal comfort are influenced not only by local meteorological variables but also by global climate phenomena. Notably, temperature increases driven by El Niño in'*Csa*'climate zones are pushing thermal stress conditions closer to those typically seen in hot desert climates, highlighting the need for city planning and heat action plans to adapt to these dynamic conditions.

The findings of this study reveal the effects of different tree species and street orientations on thermal comfort, showing that *Platanus orientalis* and *Populus canadensis* provide significant reductions in PET during morning and midday hours. However, the impact of *Robinia pseudoacacia* was found to be more inconsistent and did not result in an overall improvement in heat stress conditions. The study emphasizes that while the shading effect of trees may not entirely eliminate heat stress, it does lower PET values during certain hours, suggesting that careful selection of tree species in street planning can improve thermal comfort. Therefore, the growth rate and species selection of trees are critical elements that must be carefully considered in the context of climate change. For trees to grow healthily, factors such as groundwater levels, drought resilience, and appropriate soil conditions must be ensured. However, simply relying on vegetation to achieve thermal comfort is not sufficient. Due to the limited impact of trees, short-term solutions should also be implemented. In particular, additional strategies such as more detailed local heat risk mapping and warning systems will play a vital role in reducing heat stress. This multilayered approach will provide sustainable solutions both for improving thermal comfort and for adapting to climate change.

## Data Availability

The data that support the findings of this study are available from the corresponding author upon reasonable request.

## Appendix 1

Table 2.

**Table 2:** Hourly air temperature data during El Niño years for June, July, and August

		June										
		08:00	09:00	10:00	11:00	12:00	13:00	14:00	15:00	16:00	17:00	18:00
**2009**	**TNx**	23.8	24.3	24.9	25.4	25.9	26.2	25.9	24.7	23.6	22.3	21.4
	**TXx**	25	25.5	26	26.6	27.2	27.3	27.1	26.5	24.8	23.7	22.6
**2010**	**TNx**	23.1	23.6	23.8	23.9	24.2	24.8	24.5	23.6	22.7	21.7	21.1
	**TXx**	24.2	24.8	25.1	25.3	25.6	26	25.7	24.9	23.8	22.7	21.8
**2015**	**TNx**	22.6	22.9	23.4	23.8	23.9	24.1	23.7	22.6	21.9	20.9	20.2
	**TXx**	23.8	24.2	24.8	25.2	25.1	25.1	24.9	24	22.8	22	20.9
**2016**	**TNx**	25.1	25.7	26.3	26.7	26.6	26.5	26	25	24	22.6	21.7
	**TXx**	26.7	27.1	27.6	28.2	28.2	27.9	27.7	26.8	25.2	24	22.7
**2023**	**TNx**	24.1	24.6	25.4	25.8	26.1	25.7	25.1	24.1	22.6	21.5	20.7
	**TXx**	25.6	26.2	26.9	27.2	27.4	27.1	26.3	25.2	24	22.8	21.7

		July										
		08:00	09:00	10:00	11:00	12:00	13:00	14:00	15:00	16:00	17:00	18:00
**2009**	**TNx**	26.9	27.1	27.4	27.8	28	28.3	28.1	27	25.9	24.9	24.2
	**TXx**	27.9	28.1	28.4	28.8	29.1	29.4	29.2	28.5	27	25.9	25
**2010**	**TNx**	26.6	26.8	27.1	27.4	27.5	27.7	27.5	26.5	25.6	24.6	24.1
	**TXx**	27.8	28	28.1	28.3	28.5	28.7	28.5	27.8	26.6	25.6	24.6
**2015**	**TNx**	26.5	27	27.7	28.1	28.3	28.2	28	26.8	25.7	24.5	23.6
	**TXx**	27.7	28.2	28.7	29	29.2	29.1	29	28.3	26.9	25.7	24.4
**2016**	**TNx**	27	27.4	28.1	28.3	28.7	28.8	28.7	27.1	26	24.9	24.2
	**TXx**	28.5	28.9	29.3	29.5	29.8	29.9	29.7	29	27.1	26	24.9
**2023**	**TNx**	28.3	28.8	29.4	29.9	30.6	30.7	30.3	29.2	26.9	25.4	24.9
	**TXx**	29.4	30	30.4	31.3	31.9	32	31.6	30.9	29.4	26.9	25.6

		August										
		08:00	09:00	10:00	11:00	12:00	13:00	14:00	15:00	16:00	17:00	18:00
**2009**	**TNx**	26.2	26.9	27.4	27.6	27.7	27.2	26.5	25.4	24.3	23.7	23.2
	**TXx**	27.4	28	28.3	28.5	28.4	28.1	27.4	26.7	25.4	24.3	23.8
**2010**	**TNx**	28.9	29.2	29.7	30	30.5	30.3	29.6	28.4	27.3	26.6	26.2
	**TXx**	29.9	30.2	30.6	31.1	31.4	31.3	30.7	29.9	28.4	27.3	26.7
**2015**	**TNx**	27.6	27.8	28.2	28.6	28.8	29	28.6	27.3	26.3	25.5	24.8
	**TXx**	28.8	29	29.3	29.7	29.9	29.9	29.7	28.8	27.5	26.4	25.5
**2016**	**TNx**	27.7	28.2	28.5	28.9	28.9	28.9	28.2	26.5	25.5	24.6	24.1
	**TXx**	29.2	29.5	29.7	29.9	30.1	30.1	29.5	28.4	26.5	25.5	24.7
**2023**	**TNx**	28.5	29.4	30.1	30.6	30.9	30.5	29.4	27.9	26.5	25.8	25.5
	**TXx**	29.8	30.6	31.3	31.8	32	31.7	31.2	29.7	28	26.6	25.9

## Appendix 2

Table 3.

**Table 3:** Specifications of the Kestrel 5400

Measured parameter (°C)	Measurement range	Accuracy	Measured interval
Ta (°C)	− 29/70 °C	± 0,5 °C	1 min
RH (%)	% 0–100	± 2%	1 min
V (m/s)	0,6–40 m/s	± 3%	1 min
Globe temperature (°C)	− 29–60 °C	± 1,4 °C	1 min

## Appendix 5

Table 4.

**Table 4:** Statistical comparison of meteorological variables between PS and MS

Meteorological variable		Mean	Lower Difference	Upper Difference	Correlation	Sig
**Ta (**°C)	PS	31.2	1.2	2.1	.725	.000
	MS	29.5				
**RH (%)**	PS	45.6	− 11.9	− 8.6	.448	.000
	MS	55.8				
**V (m/s)**	PS	0.78	-.11	.06	.658	.548
	MS	0.80				

## Appendix 7 A

Table 5.

**Table 5:** Comparison of Sun and Shade Conditions Based on PET Differences Between Streets with and without Trees

*"what if"scenario*	*Platanus orientalis*						*Populus canadensis*						*Robinia pseudoaccia*					
	**EW**			**NS**			**EW**			**NS**			**EW**			**NS**		
	**L**	**C**	**R**	**L**	**C**	**R**	**L**	**C**	**R**	**L**	**C**	**R**	**L**	**C**	**R**	**L**	**C**	**R**
**LS**	*	**0.000**	*	**0.000**	**0.032**	0.280	**0.001**	**0.000**	0.100	**0.000**	**0.000**	**0.006**	*	0.151	*	**0.012**	**0.000**	*
**BS**	*	**0.000**	**0.000**	**0.000**	**0.000**	**0.000**	**0.001**	**0.000**	**0.000**	**0.000**	**0.000**	**0.001**	*	**0.005**	**0.000**	**0.009**	**0.000**	**0.030**
**RS**	*	*	**0.000**	**0.000**	**0.000**	**0.000**	*	*	**0.000**	**0.002**	**0.000**	**0.000**	*	*	**0.000**	*	*	0,098

^*^There was no change in the sun path

Results are considered statistically significant at the 95% confidence level (p < 0.05)

## Declarations

### Generative AI and AI-assisted technologies in the writing process

During the preparation of this work, the authors used ChatGPT/DeepL in order to improve language. After using this tool the authors reviewed and edited the content as needed and take full responsibility for the content of the publication.
